# Transdiagnostic clustering and network analysis for questionnaire-based symptom profiling and drug recommendation in the UK Biobank and a Korean cohort

**DOI:** 10.1038/s41598-023-49490-7

**Published:** 2024-02-24

**Authors:** Eunjin Lee, Dongbin Lee, Ji Hyun Baek, So Yeon Kim, Woong-yang Park

**Affiliations:** 1 Samsung Genome Institute, Samsung Medical Center, 81 Irwon-ro, Gangnam-gu, Seoul, 06351 Republic of Korea; 2 Department of Digital Health, Samsung Advanced Institute for Health Sciences and Technology, Sungkyunkwan University, Seoul, Republic of Korea; 3 Department of Psychiatry, Samsung Medical Center, Sungkyunkwan University School of Medicine, Seoul, Republic of Korea; 4 Department of Artificial Intelligence, Ajou University, Suwon, Republic of Korea; 5 Department of Software and Computer Engineering, Ajou University, Suwon, Republic of Korea; 6 Department of Health Science and Technology, Samsung Advanced Institute for Health Sciences and Technology, Sungkyunkwan University, Seoul, Republic of Korea; 7 Department of Molecular Cell Biology, Sungkyunkwan University School of Medicine, Suwon, Republic of Korea

**Keywords:** Computational biology and bioinformatics, Computational models, Machine learning, Predictive medicine, Health care, Diagnosis

## Abstract

Clinical decision support systems (CDSSs) play a critical role in enhancing the efficiency of mental health care delivery and promoting patient engagement. Transdiagnostic approaches that utilize raw psychological and biological data enable personalized patient profiling and treatment. This study introduces a CDSS incorporating symptom profiling and drug recommendation for mental health care. Among the UK Biobank cohort, we analyzed 157,348 participants for symptom profiling and 14,358 participants with a drug prescription history for drug recommendation. Among the 1307 patients in the Samsung Medical Center cohort, 842 were eligible for analysis. Symptom profiling utilized demographic and questionnaire data, employing conventional clustering and community detection methods. Identified clusters were explored using diagnostic mapping, feature importance, and scoring. For drug recommendation, we employed cluster- and network-based approaches. The analysis identified nine clusters using k-means clustering and ten clusters with the Louvain method. Clusters were annotated for distinct features related to depression, anxiety, psychosis, drug addiction, and self-harm. For drug recommendation, drug prescription probabilities were retrieved for each cluster. A recommended list of drugs, including antidepressants, antipsychotics, mood stabilizers, and sedative–hypnotics, was provided to individual patients. This CDSS holds promise for efficient personalized mental health care and requires further validation and refinement with larger datasets, serving as a valuable tool for mental healthcare providers.

## Introduction

The estimated global cost of mental health care is US $2.5 trillion per year, and it is expected to rise to US $6 trillion by 2030^1^. Diverse primary care system models have been implemented over the last five decades. However, issues of cost-effectiveness and accessibility to mental health care remain unresolved^2^. The proposed solutions include the continuous and updated training of primary care physicians^3^. Additionally, a recent survey found that patients’ needs for supported and shared clinical decision-making are still unmet^4^.

Artificial intelligence (AI)-based clinical decision support systems (CDSSs) may be another potential solution to address these issues^5^. While CDSSs have been widely developed in other medical fields and have improved practitioners’ performance^6^, only a few prototypical CDSSs have been provided in mental health care^7–9^. Furthermore, these prototypical CDSSs have focused only on medication choices based on their respective adverse effect profiles^7,8^. The current lack of a CDSS in mental health care may have originated from the complex and ambiguous processes involved in the diagnostic classification of psychiatric disorders, as exemplified by the Diagnostic and Statistical Manual of Mental Disorders, 5th Edition (DSM-5)^1^, as well as therapeutic decision-making heavily relies on individual clinicians’ intuition^10^.

While traditional diagnostic approaches have faced increasing criticism in recent years, transdiagnostic approaches have gained support and have provided novel insights into psychiatric research^11^. In transdiagnostic approaches, the focus shifts from the diagnosis itself to insights gained from biomarkers and raw data from psychological tests. In mental health care, aside from interventions such as psychotherapies, clinical decision-making outcomes are predominantly reflected in medication prescriptions. Medication choices in mental health care often reflect the characteristics of individual symptom profiles, regardless of the diagnosis. For instance, antipsychotics are prescribed for treating psychotic symptoms, anxiolytics for anxiety, sleep pills for insomnia, and mood stabilizers for mood fluctuations. Moreover, medication choices are adjusted based on the disease course, i.e., medication choice changes as patients transition from unipolar depression to bipolar disorder or from anxiety disorder to schizophrenia. In this regard, information regarding medication use may serve as a useful surrogate outcome reflecting disease characteristics and individual disease progression.

Alongside traditional clustering, a recent study recommended methods for community detection for application in psychiatric research, considering its complementary characteristics^12^. Community detection determines whether specific nodes are located within a network cluster together^13^. Furthermore, community detection can be advantageous for transdiagnostic approaches as it provides information regarding individual nodes and simultaneously determines the number of subgroups; moreover, it is flexible in terms of the types of similarities included^12^.

In this study, we developed a CDSS for symptom profiling and medication recommendation. We performed clustering and community detection of a network based on the demographic and mental health questionnaire data of 157,384 participants from the UK Biobank. Thereafter, we developed a system for drug recommendation based on two strategies: clustering-based and network-based. The same method was applied to a cohort of patients in a Korean tertiary hospital.

## Results

### Identifying intrinsic clusters in the UK Biobank cohort

**Figure 1:**
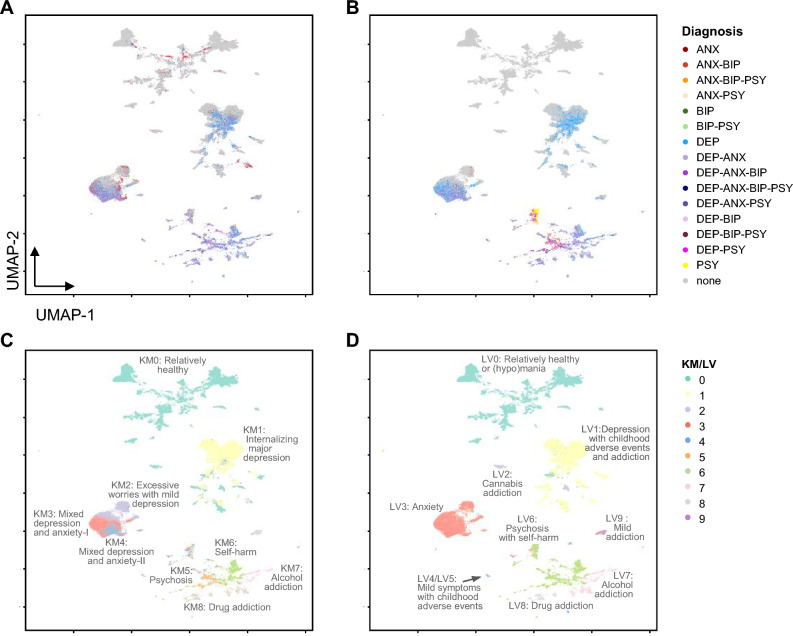
Illustration of information regarding diagnosis and results from symptom profiling based on data from 157,348 subjects using UMAP (Uniform Manifold Approximation and Projection). (**A**) Self-reported diagnosis. (**B**) Symptom-based diagnosis. (**C**) Result of k-means clustering. (**D**) Result of community detection applying the Louvain algorithm.

Characteristics of the study sample are listed in the Supplementary Fig. S1 online. Comparing the prevalence of mental illnesses between self-reported and symptom-based diagnoses, we detected inconsistencies in 33.28% of participants (see Supplementary Figs. S2 and S3 online). We performed clustering analysis based on age, sex, and questionnaire data. We identified nine and ten clusters by applying the k-means (KM) and Louvain (LV) methods, respectively (Fig. 1).

**Figure 2:**
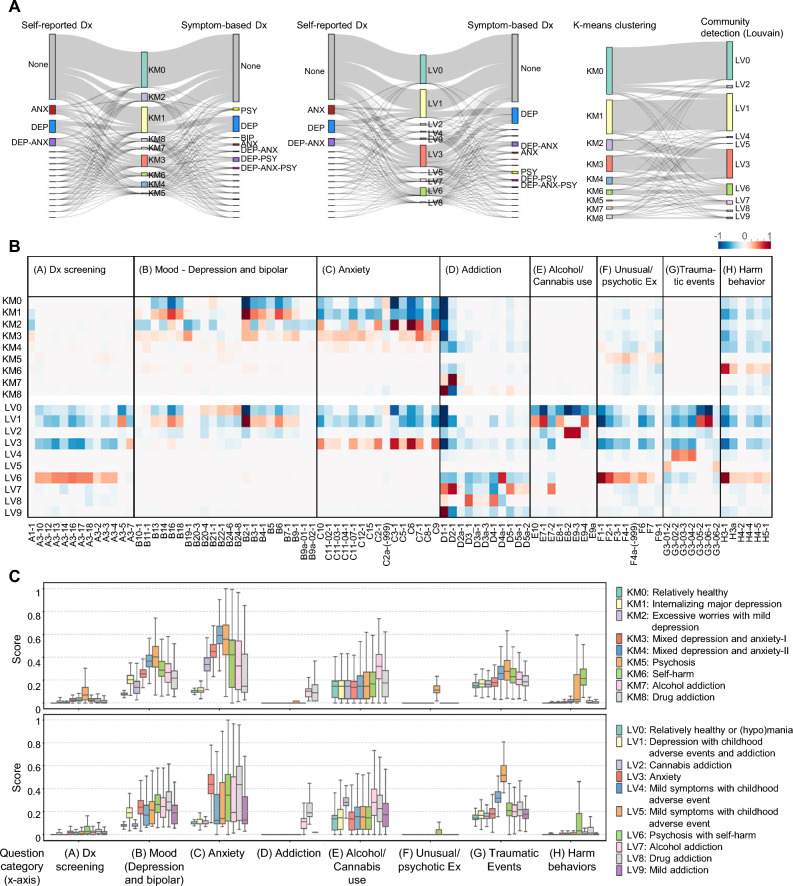
Analysis for cluster identification. (**A**) Comparison across clustering results, self-reported Dx and symptom-based Dx. Comparison between KM clustering results and Dx information (Left). Comparison between LV community detection results and Dx information (Middle). Comparison between KM clustering results and LV community detection results (Right). (**B**) Key questions identified in each cluster based on logistic regression. (**C**) Distribution of scores for the question categories in each cluster.

First, we mapped the clinical diagnostic information to the clustering results to visualize the diagnostic components within each cluster (Fig. 2A). We found that the KM0 and LV0 clusters predominantly comprised participants who were mentally healthy, whereas KM1 and LV1 clusters included participants who were relatively healthy and mildly depressed. Participants who presented with depression and anxiety symptoms were assigned to KM2, KM3, KM4, and LV3. Participants with multiple diagnoses and psychotic symptoms were included in KM5, KM6, and LV6. When comparing KM and LV clustering, the participants were most consistently assigned to KM0 and LV0, KM1 and LV1, and KM2–4 and LV3. Subsequently, we performed lasso regression to investigate the feature importance for each cluster (Fig. 2B and Supplementary Tables S5, S6 online). Items exhibiting the highest feature importance were as follows: “ever depressed for 2 weeks or more in a row” from (B) depression in KM1 and LV1; “ever worried a lot more than most people would in your situation” from (C) anxiety in KM2, KM3, KM4, and LV3; “ever seen illusions” from (F) unusual experiences in KM5 and LV6; “deliberately harmed yourself” from (H) harm behavior in KM6 and LV6; “ever addicted to alcohol” from (E) alcohol/cannabis use in KM7 and LV7; “ever addicted to one or more things, including substances or behavior” from (D) general/drug addiction in KM8 and LV9; “how often did you take cannabis” from (E) alcohol/cannabis use in LV2; “diagnosed with a life-threatening illness” from (G) traumatic events in LV4; “involved in combat or exposed to a war-zone” from (G) traumatic events in LV5; and “ever addicted to prescription or over-the-counter medicine/Illicit or recreational drugs” from (D) general/drug addiction in LV8. Furthermore, we calculated scores by summarizing the responses into eight categories of the questionnaire. Scores were considered as indicators of the level of distinct characteristics for each category (Fig. 2C). Based on the average scores for each category, the results were predominantly consistent with the feature importance patterns for most clusters. A pattern of increasing scores representing anxiety symptoms was observed from KM2 to KM3 to KM4.

Finally, considering the patterns of diagnostic mapping, feature importance, and scoring, we designated KM0 as “relatively healthy,” KM1 as “internalizing major depression,” KM2 as “excessive worries with mild depression,” KM3 and KM4 as “mixed depression and anxiety,” KM5 as “psychosis,” KM6 as “self-harm,” KM7 as “alcohol addiction,” and KM8 as “drug addiction.” For the LV result, we designated LV0 as “relatively healthy or (hypo)mania,” LV1 as “depression with childhood adverse events and addiction,” LV2 as “cannabis addiction,” LV3 as “anxiety,” LV4 and LV5 as “mild symptoms with childhood adverse events,” LV6 as “psychosis with self-harm,” LV7 as “alcohol addiction,” LV8 as “drug addiction,” and LV9 as “mild addiction”.

### Drug recommendation system in the UK Biobank cohort

Information regarding drug prescriptions was available for 14,358 participants (9.12%) of the total sample, including 154 different psychotropic drugs. The most frequently prescribed drugs in each class are summarized in the Supplementary Fig. S4 online. Among 14,358 participants, 12,526 individuals (87.2%) had been prescribed with one of the drug classes, while 1832 (12.8%) received multiple drug classes (see Supplementary Fig. S5 online). Specifically, antidepressants (AD) were used in 10,972 patients (76.42%), anti-psychotics (AP) in 421 (2.93%), mood stabilizers (MS) in 1123 (7.82%), and sedative–hypnotics (SH) in 1395 (9.72%).

**Figure 3:**
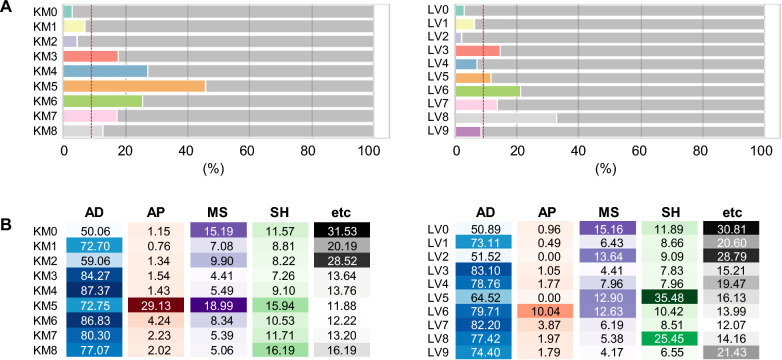
Statistics for cluster-based drug recommendation. (**A**) The percentile of samples with a history of psychiatric drugs for each cluster. The overall prescription rate across all clusters was 9.12% (red line). (**B**) The probability of prescribing each drug class to patients in a cluster.

For the cluster-based recommendations, we calculated the proportion of patients with a drug prescription history in each cluster (Fig. 3A). KM5 exhibited the highest prescription rate (45.67%), followed by KM4 (27.35%) and KM6 (25.62%). In LV clustering, LV8 exhibited the highest prescription rate (32.69%), followed by LV6 (21.24%) and LV3 (14.84%). Subsequently, the average prescription probability for each drug class was calculated and used for cluster-based recommendations (Fig. 3B). Across all clusters, AD were commonly used. With the exceptions of KM0, KM2, LV0, and LV2, the prescription probability for AD was over 70%. Users of AP were relatively concentrated in a single cluster, KM5 (29.13%) or LV6 (10.04%). In contrast, the prescription probability of MS was the highest in KM5 (18.99%), followed by KM0 (15.19%), KM2 (9.90%), and KM6 (8.34%). Regarding LV clustering, LV0 (15.15%) exhibited the highest rate of MS prescriptions, followed by LV2 (13.64%), LV5 (12.90%), and LV6 (12.63%). Finally, SH use was most common at KM8 (16.19%), KM5 (15.94%), LV5 (35.48%), and LV8 (25.45%).

**Figure 4:**
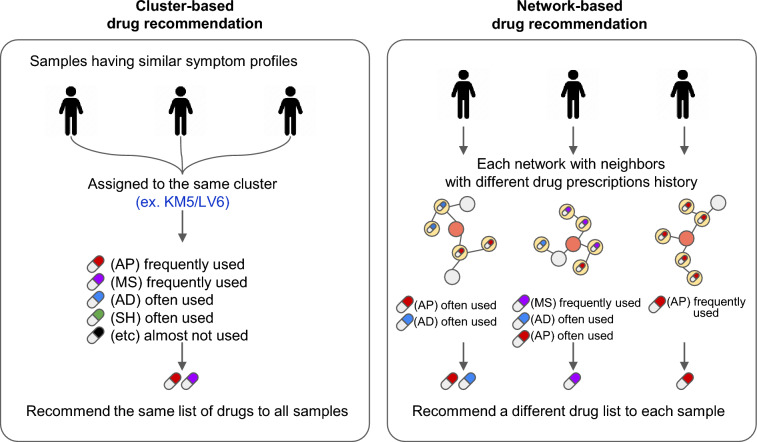
Example of drug recommendations for patients with similar symptom profiles. Cluster-based drug recommendation (Left). All samples with similar symptom profile are more likely to be assigned to the same cluster as a result of symptom profiling, such that the same list of drugs will be recommended for all samples. Network-based drug recommendation (Right). In spite of similar symptom profiles, each sample has a network of neighbors with different drug prescription histories, and a different drug list will be recommended for each sample.

Subsequently, we implemented a network-based approach that considered the prescription history of patients’ neighbors to generate a personalized list of medications that each person is most likely to be prescribed. By utilizing more local information from the network, this approach addressed individual variations within clusters, enabling a more tailored medication recommendation, even for patients with similar symptom profiles (Fig. 4). Network-based recommendations, as outlined in the Supplementary Fig. S6 online, were performed and evaluated in 14,358 patients. In cases where a tie was possible for the three most commonly recommended drugs, a single drug class was recommended for 6211 samples (43.26%), two drug classes for 5876 (40.92%), three drug classes for 1619 (11.28%), four drug classes for 497 (3.46%), and all five to 155 (1.08%). Considering the different drug classes, AD were recommended for 14,303 samples (99.62%), AP for 517 (3.6%), MS for 1,839 (12.81%), and SH for 1,744 (12.15%). Combined with the clustering results, AD were commonly recommended across all clusters (see Supplementary Fig. S7 online). As in the actual prescription data, AP were predominantly recommended to patients in the KM5 and LV6 groups. MS were highly recommended for KM0, KM5, and LV0, but to a lesser extent for LV5 compared to the actual treatment data. SH were predominantly recommended for patients in KM8, LV5, and LV8.

### Analysis of the Korean cohort

The Korean cohort, comprising psychological and prescription data from 1307 patients, was obtained from the Samsung Medical Center (SMC), a tertiary hospital in Seoul, Korea. The analysis focused on 842 patients without missing values. The diagnoses of patients in the Korean cohort based on the DSM-5 (DxP) and scale (DxS) are summarized in the Supplementary Fig. S8 online. Regarding diagnoses by DxP, patients with only depression (37.89%) were most common, followed by those with only anxiety (19.83%). For diagnoses by DxS, patients with depression, anxiety, and bipolar disorder (43.35%) were most common, followed by patients with depression and anxiety (40.14%).

Drug prescription data were available for 94.3% patients in the cohort (see Supplementary Fig. S9 online). Considering the number of prescribed drug classes, 31.6%, 39.6%, and 23.2% patients were prescribed one, two, and three or more classes, respectively. The prescription probabilities per drug class were 76.38% for AD, 25.94% for AP, 13.48% for MS, and 63.1% for SH.

**Figure 5:**
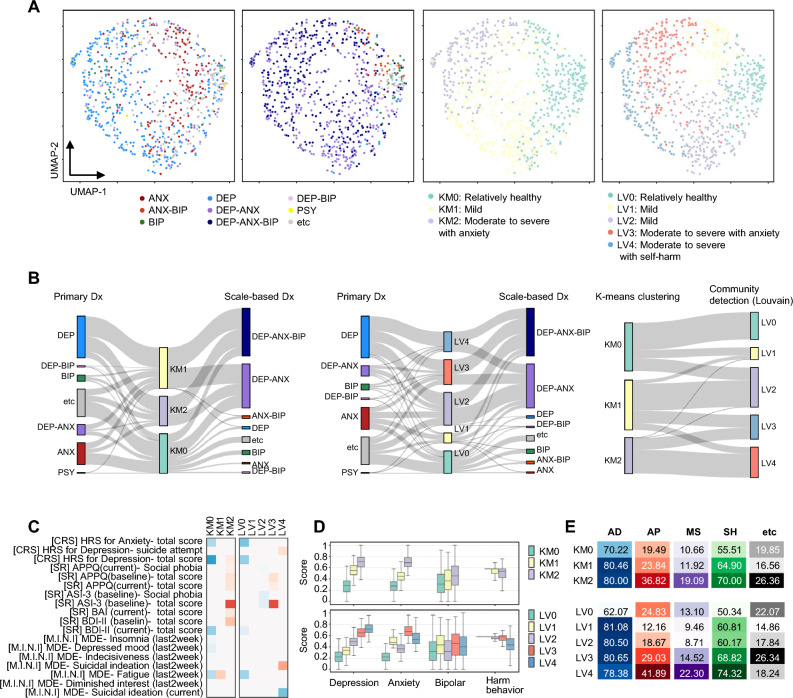
Analysis of the Korean cohort. (**A**) Illustration of information on diagnosis and results from symptom profiling of 842 patients using UMAP. (From left to right) Primary diagnosis; Scale-based diagnosis; Result of k-means clustering; Result of community detection applying Louvain algorithm. (**B**) Comparison across clustering results, Primary Dx and Scale-based Dx. (**C**) Key questions in each cluster as a result of logistic regression. *Abbreviations. CRS: Clinical Rating Scale; HRS: Hamilton Rating Scale; SR: Self-report; APPQ: Albany Panic and Phobia Questionnaire; ASI: Anxiety Sensitivity Index-3; BAI: Beck Anxiety Inventory; BDI: Beck Depression Inventory-II; MINI: Structured Interview; MDE: Major Depressive Episode. (**D**) Distribution of scores for the question categories in each cluster. **(E**) The probability of prescribing each drug class to patients in a cluster.

Three and five clusters were identified using the KM and LV methods, respectively (Fig. 5A). The results, including diagnosis mapping, feature importance patterns, and scores for each item, are presented in Fig. 5B–D. The clustering results reflected the severity of symptoms rather than symptom profiles; hence, we designated SMC-KM0 as “relatively healthy,” SMC-KM1 as “mild,” and SMC-KM2 as “moderate to severe anxiety.” Similarly, SMC-LV0 was named as “relatively healthy,” SMC-LV1 and SMC-LV2 as “mild,” SMC-LV3 to “moderate to severe anxiety,” and SMC-LV4 as “moderate to severe self-harm”.

The overall rate of drug prescriptions did not differ significantly between clusters, ranging from 84.8–97.37% (see Supplementary Fig. S10 online). In both the KM and LV clusters, the AD prescription rate was lower in KM0 and LV0 than in the other clusters (Fig. 5E). AP, MS, and SH generally exhibited patterns of increasing prescription rates from KM0 to KM2 and LV1 to LV4. AP or MS prescription rates were higher for LV0 than for LV1 or LV2. This was consistent with the results from network-based recommendation (see Supplementary Fig. S11 online).

## Discussion

Our study contributes to the growing interest in the application of AI-based CDSS in mental health care^14–16^. A recent study evidenced the potential of a CDSS with high diagnostic accuracy using various machine-learning models for diagnosing multiple mental disorders^14^. For this, researchers employed a Network Pattern Recognition (NEPAR) algorithm to simplify the questionnaire and trained prediction models to anticipate the presence and type of mental disorders. In contrast, our methodology relied exclusively on the symptoms outlined in the questionnaire, avoiding pre-existing diagnostic information as the basis for correct answers. This data-driven approach revealed diverse classification possibilities that were previously obscured by existing diagnostic information. Moreover, drug recommender systems have been developed to aid end-users and health care professionals in identifying appropriate medications for specific diseases or even multi-disease cases^17,18^. Although various studies share similarities with ours in identifying similar individuals based on attributes, they differ in their methods, each employing different algorithms and measures to define similarity. Our approach uniquely performs symptom-based network analysis to identify the most similar neighbors. This has allowed us to detect clusters of co-occurring symptoms, offer deeper insights into the fundamental mechanisms of mental disorders and aiding in treatment decisions. This approach reveals the increased complexity of patient conditions through strong connectivity^19,20^. As mentioned, developing similar systems for mental health disorders has been challenging due to the complexity and variability of mental disorders, the lack of objective biomarkers, and individual differences in drug response^7–10,21^. Despite these challenges, the proposed system offers a promising alternative for navigating the intricacies involved in recommending mental health medications.

In this study, the k-means clustering and Louvain methods have different advantages in identifying clusters. In KM clustering, patients exhibiting depressive symptoms were distributed into multiple clusters according to the accompanying symptom profiles, such as worries, anxiety, or externalizing behaviors. Additionally, patients exhibiting self-harm and those presenting with psychotic symptoms tended to cluster together, although they had different symptom profiles. In the LV clustering, addiction symptoms and childhood adverse events had a relatively strong influence on the clustering results.

Furthermore, we propose two types of drug recommendation systems with different strengths. The cluster-based recommendation provided the “most commonly prescribed drug in a cluster,” which may be a safer strategy because this may reflect the whole cluster’s characteristics. In contrast, the network-based method helped make more detailed recommendations because it focuses more on small communities, called neighborhoods, and considers local characteristics. For drug recommendations for the UK Biobank cohort, AD recommendation was particularly limited due to their high prescription rate. Use of AP was concentrated in clusters predominantly indicating psychosis (KM5 and LV6), Suggesting that AP users tend to cluster around psychotic symptom profiles. Notably, the rate of MS use was more broadly distributed than that of AP use. MS use was most common in KM5 (“psychosis,” 18.99%), followed by KM0 (“relatively healthy,” 15.19%), KM2 (“excessive worries with mild depression,” 9.90%), and KM6 (“self-harm,” 8.34%,). This may indicate that MS was prescribed to patients with multiple comorbidities or severe symptoms, such as self-harm, and to those possibly suggesting some degree of bipolar disorder. SH were predominantly used in KM8 (“drug addiction”) and LV8 (“drug addiction”), which could imply SH abuse. Additionally, LV5 (“Mild symptoms with childhood adverse events”) was notably linked to a high rate of SH use, though the underlying reason remains unclear.

The SMC cohort analysis presented results that were different from the UK Biobank data. The clustering results were heavily affected by overall symptom severity rather than by detailed symptom profiles. This may be explained by various characteristics of the cohort. First, the SMC cohort included only patients visiting a tertiary psychiatric clinic, and excluded those with minimal to mild severity from the general population. An easier access to tertiary hospitals in Korea may also be related to the different results compared with the UK Biobank data. Second, psychological evaluations were designed and prescribed predominantly in outpatient clinics for patients with a seemingly low probability of psychosis. Finally, the different ethnicities of the two cohorts may have contributed to differences in the results.

Our findings have important implications for transdiagnostic approaches and drug recommendation systems for mental health. To our knowledge, we are the first to demonstrate the utility of transdiagnostic clustering and a network-based approach using psychological questionnaire data for drug recommendation in mental health. Furthermore, the novel clusters identified in this study can serve as endophenotypes for the discovery of genomic and other biological markers. Moreover, the identification of specific psychological items with high importance, derived from feature importance analysis, provides a basis for simplifying the questionnaires used for transdiagnostic clustering. By identifying and emphasizing these critical psychological items, our study suggests a means of potentially refining and simplifying questionnaires employed in similar analytical procedures. Specifically, our study suggests that the target group of the medical delivery system—the general population in the UK Biobank cohort and patients visiting tertiary hospitals in the SMC cohort—must be considered when developing a CDSS.

Despite being based on a comprehensive analysis, this study has some limitations. First, we could not provide performance evaluation metrics for the drug recommendation systems. As the SMC data had a profile of samples and questionnaire data that differed from the UK Biobank data, they could not be used for validation or evaluation purposes. Second, there is potential for significant algorithm enhancement. This study primarily introduces a new approach to questionnaire-based symptom profiling and drug recommendations; however, a more extensive exploration of sophisticated methodologies was not conducted. Future research could benefit from exploring a broader array of methodologies and fine-tuning relevant parameters to achieve more accurate and precise outcomes. Regarding drug recommendation algorithms, addressing variance in drug use frequency, particularly the active recommendation of AD due to their high prescription frequency, is crucial. Future studies should consider a hybrid approach that covers both global and local characteristics by combining cluster- and network-based approaches. Additionally, adding the option “no prescription of any drug” to the recommendation results may be beneficial.

In conclusion, this study identified transdiagnostic clusters in mental health using conventional clustering and network community detection based on questionnaire data. Furthermore, we propose a drug recommendation system that uses clustering- and network-based methods. The intrinsic transdiagnostic clusters and patterns of medication use observed in this study can be utilized for further discovery of biomarkers. After further validation and elaboration using larger datasets, the CDSS may provide tools for efficient personalized medicine in mental health care.

## Methods

### The UK Biobank cohort

The UK Biobank is a prospective cohort of over 500,000 participants that provides information on health status, lifestyle behavior, family history, and sociodemographics^22^. For mental health research, an online questionnaire was developed by an expert working group funded by the UK Biobank, and data were collected between 2016 and 2017 from participants who received an email invitation or were surveyed via a participant website^23^. The questionnaire comprised the World Health Organization Composite International Diagnostic Interview (CIDI)^24^ and complementary instruments that are widely used and established in mental health research. Detailed information is available on the UK Biobank website (http://biobank.ctsu.ox.ac.uk/crystal/refer.cgi?id=22). The questionnaire covered lifetime experiences in mental health and addressed the following topics: (A) diagnostic screening, (B) mood disorder (depression and bipolar), (C) anxiety disorder, (D) general/drug addiction, (E) alcohol and cannabis use, (F) unusual and psychotic experiences, (G) traumatic events in life, and (H) harmful behaviors. Overall, 141 questions were used (see Supplementary Table S1 online). We used two types of diagnostic (Dx) information from the participants. First, we used self-reported diagnosis (“Have you been diagnosed with mental health problems by a professional, even if you do not have it currently?” UK Biobank field id #20544). Second, we used a score-based diagnosis by modifying the criteria from a comparative study of four different indicators of psychiatric disorders using the UK Biobank in 2019 (see Supplementary Table S2 online)^25^.

In this study, web-based questionnaire data on mental health from 157,348 respondents were used for the clustering analysis. Data on medication use based on self-reports, available from 14,358 respondents (UK Biobank field id #20003), were included for drug recommendations. After pre-processing, including the correction of typos and removal of duplicates, 154 psychotropic drugs were selected through manual curation by a psychiatrist and classified into four different drug classes: antidepressants (AD), antipsychotics (AP), mood stabilizers (MS), and sedative–hypnotics (SH) drugs (see Supplementary Tables S3, S4 online).

This study was conducted using the UK Biobank resource (application number: 33002). The UK Biobank study received ethical approval from the NHS National Research Ethics Service and has approval from the North West Multi-Centre Research Ethics Committee (11/NV/0382). All procedures were performed in accordance with the relevant guidelines and regulations.

### Clustering analysis

We performed clustering analysis to define distinct subgroups based on questionnaire data from 157,348 respondents in the UK Biobank cohort. The analyses were conducted using two methods: k-means clustering and network-based community detection. In k-means clustering, objects are divided into k clusters, with the objects belonging to the cluster having each cluster centroid^26^. The Euclidean distance measure, chosen for its compatibility with continuous and numerical features, computational ease, and capability of spatial associations among data points, was used as the similarity function^27^. For network-based community detection, we first constructed a network using the k-nearest neighbor algorithm, wherein nodes represented each sample and edges indicated the similarity between samples based on symptoms and history of mental illness^28^. We took 100-nearest neighbors for each node to strike a balance between representativeness and computational feasibility. We utilized the Minkowski distance measure with p = 2 to enable a standard Euclidean distance metric, thereby compatible with k-means clustering^29^. Subsequently, we applied the Louvain algorithm, which is one of the fastest and most popular methods for community detection^30^. The Louvain algorithm optimizes the modularity of a network by repeatedly performing community building within it^31,32^.

Overall, 141 questionnaire items including age and sex were included in the data analysis. Age information was self-reported, and sex information included a combination of sex recorded by the National Health Service (NHS) and self-reported sex. We applied one-hot encoding to the categorical variables and removed ambiguous variables that indicated missing values. A total of 268 features were available for analysis. Standard scaling was applied for normalization. Clustering analysis was implemented by changing the number of clusters and resolution of the k-means and Louvain algorithms. A silhouette score was used to determine the optimal number of clusters^33^. To visualize the clustering results, we performed a Uniform Manifold Approximation and Projection, a nonlinear dimension reduction algorithm, and an effective tool for visualizing clusters with their relative proximities^34^.

These clusters were further investigated for clinical significance. First, clustering results were mapped to diagnostic information using a Sankey diagram to visualize correlations through the proportion of overlapping^35^. Second, lasso regression was performed on all questionnaire items, identifying features with significant importance, as indicated by a beta coefficient greater than 0.2 (Supplementary Tables S5–S6 online)^36,37^. Finally, index scores for each questionnaire item category (A)–(H) were computed to examine feature importance. The data was normalized using min–max scaling, and responses were summarized by averaging values obtained within each category.

### Development of drug recommendation systems

We developed two drug recommendation systems: clustering-based and network-based. In the clustering approach, we calculated drug use frequency within each cluster, providing insights into the likelihood of each drug class (AD, AP, MS, and SH) being prescribed. For the network-based approach, we utilized a network generated for community detection in patient clustering, and created a list of medications that each person was most likely to be prescribed considering their neighbors’ prescription histories (see Supplementary Fig. S6 online). The execution steps were as follows: (i) Establish the initial neighborhood by selecting individuals most similar to a target patient within the pre-constructed network, generated from community detection covering 157,348 individuals. Because only approximately 9% (14,358) individuals had accessible information regarding drug prescriptions, some of these neighbors may have information on drug prescriptions, while others may not. (ii) Expand the neighborhood gradually, to ensure sufficient drug prescription data within the neighborhood, requiring at least N individuals with accessible drug prescription information. In this study, we set this number to 20 individuals, and this criterion is adjustable and depends on the severity of mental illness and availability of drug prescription history. (iii) Finalize drug recommendations based on the information obtained from the final neighborhood, compiling a list of recommended drugs ordered by prescription frequency. The final recommendations comprised drug classes with prescription rankings exceeding a predetermined threshold.

Thereafter, the predicted drug classes were compared with those reported to be prescribed to each individual. Furthermore, we calculated the recommendation frequency for each drug class in each cluster.

### Korean cohort of the Samsung Medical Center (SMC)

The Korean data comprised psychological evaluations and prescription information of 1307 patients who visited the psychiatry department of the SMC, a university-affiliated tertiary hospital in Seoul, Korea. Psychological evaluation data included 318 items from structural interviews, clinical rating scales, and self-reported scales. This assessment, named the “Adult Screening Psychological Evaluation”, was primarily administered during the initial screening of patients with a low probability of psychosis. The evaluation battery excluded items for a detailed assessment of psychotic symptoms. Among these items, 80 questions with the fewest missing responses (see Supplementary Table S7 online) were included in the analysis, involving 842 patients without missing data. The questionnaire data included 20 questions from a structural interview (MINI), 33 questions from the Clinician Rating Scales (Hamilton Depression Rating Scale [HAMD], Hamilton Anxiety Rating Scale [HAMA], and Panic Disorder Severity Scale [PDSS]), and 27 questions from self-reported scales.

The diagnosis was determined based on two strategies: DSM-based primary diagnosis (DxP) and scale-based diagnosis (DxS). DxP was assigned by psychologists within the framework of clinical psychological assessments according to the hierarchical diagnostic system of the DSM-5 criteria. Regarding DxS, depression was defined as a total score of 7 or higher on the HAMD, anxiety disorder was diagnosed when the total score on the HAMA was 10 or higher or the total score on the PDSS was 8 or higher, and bipolar disorder was diagnosed when the self-reported Mood Disorder Questionnaire (MDQ) score was 7 or higher or the Hypomania Check List (HCL)-32 score was 12 or higher. No scale measuring psychotic symptoms was used in this study. Thereafter, SMC data, including self-reported age and sex, were analyzed using the same methods as those described for the aforementioned UK Biobank cohort.

The study protocol was approved by the Institutional Review Board (IRB) of the Samsung Medical Center (IRB SMC no. 2018-11-019). The IRB Samsung Medical Center (IRB SMC no. 2018-11-019) waived the requirement for written informed consent because this study was a retrospective chart review, and all identifying data were removed from the clinical database prior to analysis. We confirmed that the procedures followed all applicable guidelines and regulations to respect patient privacy, maintain scientific integrity, and meet the necessary ethical standards.

### Supplementary Information

Supplementary Information 1.

Supplementary Table S6.

## Data Availability

Data from UK Biobank are available to researchers upon making an application (http://www.ukbiobank.ac.uk/using-the-resource/). Data from Samsung Medical Center are available upon reasonable request to the corresponding author.
