# Supplementary material for: Transdiagnostic clustering and network analysis for questionnaire-based symptom profiling and drug recommendation in the UK Biobank and a Korean cohort
Source: Sci Rep. 2024 Feb 24;14:4500. doi: 10.1038/s41598-023-49490-7 (PMC10894302; doi:10.1038/s41598-023-49490-7)
Supplement: Supplementary file 1 — Supplementary Information 1. [file 41598_2023_49490_MOESM1_ESM.docx]

**Transdiagnostic clustering and network analysis for questionnaire-based symptom profiling and drug recommendation in the UK Biobank and a Korean cohort**

Authors

Eunjin Lee^†^, Dongbin Lee^†^, Ji Hyun Baek, So Yeon Kim, and Woong-yang Park*

^†^ These authors contributed equally to this work.

* Send correspondence to Dr. Woong-Yang Park ([woongyang.park@samsung.com](mailto:woongyang.park@samsung.com))

Samsung Genome Institute, Samsung Medical Center, 81 Irwon-ro, Gangnam-gu, Seoul 06351, Republic of Korea

**Supplementary information**

**Table S1.** Number of questions in each category

**Table S2.** Score-based Dx Criteria for mental disorders

**Table S3.** Number of psychotropic drugs in each drug class

**Table S4.** List of psychotropic drugs (n = 154)

**Table S5.** The number of questions which showed significance as important features in each cluster

**Table S6.** A full list of significant questions from each cluster

**Table S7.** SMC questionnaire for analysis (n = 80)

**Figure S1.** Characteristics of UKB cohort

**Figure S2.** Prevalence of mental disorders by self-reported diagnosis and symptom-based diagnosis

**Figure S3.** Distribution of disease combinations and comparison between self-reported Dx and symptom-based Dx

**Figure S4.** Frequency of the most frequently prescribed drugs in each drug class

**Figure S5.** Drug prescription information of 14,358 individuals in UKB cohort

**Figure S6.** Overview of the network-based recommendation system

**Figure S7.** Results of network-based drug recommendation with cluster information

**Figure S8.** Distribution of disease combination and comparison between self-reported Dx and symptom-based Dx in SMC cohort

**Figure S9.** Drug prescription information in SMC cohort

**Figure S10.** Statistics for cluster-based drug recommendation in SMC cohort

**Figure S11.** Results of network-based drug recommendation including cluster information

**Table S1.** Number of questions in each category

| **Question category** | **N** |
| --- | --- |
| Total | 141 |
| A. Mental distress (Dx Screening) | 3 |
| B. Mood disorder: Depression/Mania | 37 |
| C. Anxiety disorder | 28 |
| D. Addictions | 12 |
| E. Alcohol/Cannabis use | 11 |
| F. Unusual and psychotic experiences | 14 |
| G. Traumatic events | 21 |
| H. Self-harm behaviors | 10 |
| J. Happiness and subjective well-being | 3 |

**Table S2.** Score-based Dx Criteria for mental disorders

| **Diagnosis** | **symptom** | **Criteria** |
| --- | --- | --- |
| **Depression** | **Case: Depression ever.**  At least one core symptom of depression, for most of the day or during the whole day, on most or all days for a two week period, with at least five depressive symptoms that represent a change from the usual state over the same time period, with some or significant impairment. | [‘AND’ for all conditions]   - Persistent sadness (20446) = Yes OR Loss of interest (20441) = Yes - How much of day (20436) = Most of day or All day long (3 or 4) - Did you feel this way (20439) = Almost every day or Every day (2 or 3) - Impairment (20440) = Somewhat or A lot (2 or 3) - Total number of symptoms endorsed (core and others) >= 5 Persistent sadness (core) 20446; Loss of interest (core) 20441; Tired or low energy 20449; Gain or loss of weight 20536 (1,2,3) = Gain, Loss, or Gain and loss; Sleep change 20532; Trouble concentrating 20435; Feeling worthless 20450; Thinking about death 20437 |
| **Anxiety disorder** | **Case: GAD Ever.**  Excessive worrying regarding numerous issues, occurring on most days for six months, and that are difficult to control, with three or more somatic symptoms and functional impairment. | [‘AND’ for all conditions]   - Worried, tense, or anxious (20421) = Yes - Duration (20420) >= 6 months or All my life (;-999) - Most days (20538) = Yes - Excessive: More than most (20425) OR Stronger than most (20542) - Number of issues: More than one thing (20543; 2) OR Different worries (20540) - Difficult to control: Difficult to stop worrying (20541) OR Couldn’t put it out of mind (20539; 3) OR Difficult to control (20537; 3) - Functional impairment: Role interference (20418) = Some or A lot (2,3) - 3 somatic symptoms out of: Restless. 20426; Keyed up or on edge. 20423; Easily tired. 20429; Having difficulty keeping your mind on what you were doing. 20419; More irritable than usual. 20422; Having tense, sore, or aching muscles. 20417; Frequently having trouble falling or staying asleep. 20427 |
| **Bipolar disorder** | **Symptoms: Hypomania / Mania.**  Endorses features of hypomania / mania lasting for a week or more, whether or not they are disruptive, and with or without a history of depression. Requires “mania” plus three other symptoms or “Irritable” plus four other symptoms | [‘AND’ for all conditions]   - High/Hyper 20501 = 01 OR Irritable 20502 = 01 - Four features from: High/Hyper 20501; Active 20548(01); Talkative 20548(02); Less sleep 20548(03); Creative/ideas 20548(04); Restless 20548(5); Confident 20548(6); Thoughts racing 20548(7); Easily distracted 20548(8) - Duration 20492 = A week or more (3) |
| **Psychotic disorder including schizophrenia** | **Symptoms: Psychotic experience.**  Endorsed possible hallucination or delusion | [‘OR’ for all conditions]   - Heard unreal voice 20463 = yes - Saw unreal vision 20471 = yes - Believed unreal conspiracy 20468 = yes - Believed unreal communication or signs 20474 = yes |

**Table S3.** Number of psychotropic drugs in each drug class

| **Drug class*** | **N** |
| --- | --- |
| Total | 154 |
| AD: Antidepressant | 55 |
| AP: Antipsychotics | 44 |
| MS: Mood stabilizer | 13 |
| SH: Sedative hypnotic | 26 |
| etc | 16 |

*curated by clinician

**Table S4.** List of psychotropic drugs (n = 154)

|  | **Drug class*** | **Drug name** | **UKB code** |
| --- | --- | --- | --- |
| 1 | AD | citalopram | 1140921600 |
| 2 | AD | fluoxetine | 1140879540 |
| 3 | AD | amitriptyline | 1140879616 |
| 4 | AD | sertraline | 1140867878 |
| 5 | AD | venlafaxine | 1140916282 |
| 6 | AD | paroxetine | 1140867888 |
| 7 | AD | mirtazapine | 1141152732 |
| 8 | AD | dosulepin | 1140909806 |
| 9 | AD | escitalopram | 1141180212 |
| 10 | AD | seroxat 20mg tablet | 1140882236 |
| 11 | AD | prozac 20mg capsule | 1140867876 |
| 12 | AD | trazodone | 1140879634 |
| 13 | AD | st john's wort/hypericum [ctsu] | 1201 |
| 14 | AD | duloxetine | 1141200564 |
| 15 | AD | cipralex 5mg tablet | 1141190158 |
| 16 | AD | lofepramine | 1140867726 |
| 17 | AD | clomipramine | 1140879620 |
| 18 | AD | efexor 37.5mg tablet | 1140916288 |
| 19 | AD | nortriptyline | 1140867818 |
| 20 | AD | imipramine | 1140879630 |
| 21 | AD | cipramil 10mg tablet | 1141151946 |
| 22 | AD | dothiepin | 1140879628 |
| 23 | AD | prothiaden 25mg capsule | 1140867624 |
| 24 | AD | reboxetine | 1141151978 |
| 25 | AD | lustral 50mg tablet | 1140867884 |
| 26 | AD | trimipramine | 1140867756 |
| 27 | AD | zispin 30mg tablet | 1141152736 |
| 28 | AD | amitriptyline hydrochloride+perphenazine 10mg/2mg tablet | 1140867948 |
| 29 | AD | cymbalta 30mg gastro-resistant capsule | 1141201834 |
| 30 | AD | anafranil 10mg capsule | 1140867690 |
| 31 | AD | moclobemide | 1140867920 |
| 32 | AD | phenelzine | 1140867850 |
| 33 | AD | fluvoxamine | 1140879544 |
| 34 | AD | tranylcypromine | 1140867914 |
| 35 | AD | surmontil 10mg tablet | 1140867758 |
| 36 | AD | doxepin | 1140867640 |
| 37 | AD | triptafen tablet | 1140867934 |
| 38 | AD | yentreve 20mg gastro-resistant capsule | 1141200570 |
| 39 | AD | nardil 15mg tablet | 1140867852 |
| 40 | AD | edronax 4mg tablet | 1141151982 |
| 41 | AD | allegron 10mg tablet | 1140867820 |
| 42 | AD | mianserin | 1140879556 |
| 43 | AD | faverin 50mg tablet | 1140867860 |
| 44 | AD | tranylcypromine+trifluoperazine 10mg/1mg tablet | 1140867944 |
| 45 | AD | molipaxin 50mg capsule | 1140882244 |
| 46 | AD | nefazodone | 1140917460 |
| 47 | AD | gamanil 70mg tablet | 1140882310 |
| 48 | AD | amitriptyline+chlordiazepoxide 12.5mg/5mg capsule | 1140867938 |
| 49 | AD | manerix 150mg tablet | 1140867922 |
| 50 | AD | maoi - tranylcypromine | 1140910820 |
| 51 | AD | limbitrol 10 capsule | 1140856186 |
| 52 | AD | norval 10mg tablet | 1140867812 |
| 53 | AD | tofranil 10mg tablet | 1140867712 |
| 54 | AD | amoxapine | 1140867774 |
| 55 | AD | sinequan 10mg capsule | 1140882312 |
| 56 | AP | olanzapine | 1140928916 |
| 57 | AP | quetiapine | 1141152848 |
| 58 | AP | risperidone | 1140867444 |
| 59 | AP | chlorpromazine | 1140879658 |
| 60 | AP | trifluoperazine | 1140868120 |
| 61 | AP | amisulpride | 1141153490 |
| 62 | AP | seroquel 25mg tablet | 1141152860 |
| 63 | AP | sulpiride | 1140867304 |
| 64 | AP | aripiprazole | 1141195974 |
| 65 | AP | haloperidol | 1140867168 |
| 66 | AP | stelazine 1mg tablet | 1140867244 |
| 67 | AP | depixol 3mg tablet | 1140867152 |
| 68 | AP | clozapine | 1140867420 |
| 69 | AP | flupentixol | 1140909800 |
| 70 | AP | promazine | 1140879746 |
| 71 | AP | fluanxol 500micrograms tablet | 1140867952 |
| 72 | AP | risperdal 0.5mg tablet | 1141177762 |
| 73 | AP | modecate 12.5mg/0.5ml oily injection | 1140867456 |
| 74 | AP | zyprexa 2.5mg tablet | 1141167976 |
| 75 | AP | flupenthixol | 1140867150 |
| 76 | AP | zuclopenthixol | 1140882100 |
| 77 | AP | largactil 10mg tablet | 1140863416 |
| 78 | AP | haldol 5mg tablet | 1140867184 |
| 79 | AP | abilify 5mg tablet | 1141202024 |
| 80 | AP | clopixol 2mg tablet | 1140867342 |
| 81 | AP | clozaril 25mg tablet | 1140882320 |
| 82 | AP | cpz - chlorpromazine | 1140910358 |
| 83 | AP | fluphenazine | 1140882098 |
| 84 | AP | pericyazine | 1140867134 |
| 85 | AP | fentazin 2mg tablet | 1140867210 |
| 86 | AP | fluphenazine decanoate | 1140867398 |
| 87 | AP | dolmatil 200mg tablet | 1140867306 |
| 88 | AP | levomepromazine | 1140909802 |
| 89 | AP | perphenazine | 1140867208 |
| 90 | AP | zaponex 25mg tablet | 1141201792 |
| 91 | AP | neulactil 2.5mg tablet | 1140867136 |
| 92 | AP | zotepine | 1141169714 |
| 93 | AP | serenace 500micrograms capsule | 1140867092 |
| 94 | AP | thioridazine | 1140879750 |
| 95 | AP | denzapine 25mg tablet | 1141200458 |
| 96 | AP | pimozide | 1140867218 |
| 97 | AP | fluphenazine hydrochloride+nortriptyline 1.5mg/30mg tablet | 1140867940 |
| 98 | AP | benperidol | 1140867078 |
| 99 | AP | sertindole | 1140927956 |
| 100 | MS | lithium product | 1140867490 |
| 101 | MS | priadel 200mg m/r tablet | 1140867504 |
| 102 | MS | lamotrigine | 1140872290 |
| 103 | MS | tegretol 100mg tablet | 1140872072 |
| 104 | MS | sodium valproate | 1140872198 |
| 105 | MS | carbamazepine | 2038459704 |
| 106 | MS | epilim 100mg crushable tablet | 1140872200 |
| 107 | MS | depakote 250mg e/c tablet | 1141172838 |
| 108 | MS | topiramate | 1140923484 |
| 109 | MS | valproic acid | 1140872214 |
| 110 | MS | lamictal 25mg tablet | 1140872302 |
| 111 | MS | camcolit 250 tablet | 1140867494 |
| 112 | MS | carbamazepine product | 1140872064 |
| 113 | SH | zopiclone | 1140863144 |
| 114 | SH | diazepam | 1140863152 |
| 115 | SH | temazepam | 1140863202 |
| 116 | SH | zolpidem | 1140865016 |
| 117 | SH | clonazepam | 1140872150 |
| 118 | SH | nitrazepam | 1140863182 |
| 119 | SH | lorazepam | 1140863302 |
| 120 | SH | zimovane ls 3.75mg tablet | 1140928004 |
| 121 | SH | valium 2mg tablet | 1140863244 |
| 122 | SH | oxazepam | 1140863442 |
| 123 | SH | chlordiazepoxide | 1140863328 |
| 124 | SH | clobazam | 1140863268 |
| 125 | SH | loprazolam | 1140863120 |
| 126 | SH | stilnoct 5mg tablet | 1140864916 |
| 127 | SH | xanax 250mcg tablet | 1140863310 |
| 128 | SH | alprazolam | 1140863308 |
| 129 | SH | diazepam product | 1141157496 |
| 130 | SH | zaleplon | 1141171404 |
| 131 | SH | sonata 5mg capsule | 1141171410 |
| 132 | SH | bromazepam | 1140863318 |
| 133 | SH | ativan 1mg tablet | 1140863364 |
| 134 | SH | medazepam | 1140863372 |
| 135 | SH | valium 10mg suppository | 1140855856 |
| 136 | SH | mogadon 5mg tablet | 1140863194 |
| 137 | SH | rohypnol 1mg tablet | 1140863106 |
| 138 | SH | flurazepam | 1140863110 |
| 139 | etc | propranolol | 1140879842 |
| 140 | etc | gabapentin | 1140872228 |
| 141 | etc | pregabalin | 1141200004 |
| 142 | etc | procyclidine | 1140883476 |
| 143 | etc | clonidine | 1140883468 |
| 144 | etc | ropinirole | 1140928274 |
| 145 | etc | nicotine product | 1140872492 |
| 146 | etc | buspirone | 1140879730 |
| 147 | etc | inderal 10mg tablet | 1140866804 |
| 148 | etc | clonidine hydrochloride 25micrograms tablet | 1140871986 |
| 149 | etc | trihexyphenidyl | 1140909816 |
| 150 | etc | bupropion | 1141176854 |
| 151 | etc | buspar 5mg tablet | 1140863454 |
| 152 | etc | donepezil hydrochloride | 1141150834 |
| 153 | etc | atomoxetine | 1141199446 |
| 154 | etc | rivastigmine | 1141167690 |

*Abbreviations
AD: antidepressant
AP: anti-psychotics
MS: mood stabilizer
SH: sedative-hypnotics

**Table S5.** The number of questions which showed significance as important features in each cluster.

|  | KM0 | KM1 | KM2 | KM3 | KM4 | KM5 | KM6 | KM7 | KM8 |  |
| --- | --- | --- | --- | --- | --- | --- | --- | --- | --- | --- |
| num.Q | 21 | 27 | 25 | 17 | 9 | 1 | 7 | 1 | 2 |  |
|  | LV0 | LV1 | LV2 | LV3 | LV4 | LV5 | LV6 | LV7 | LV8 | LV9 |
| num.Q | 45 | 51 | 8 | 38 | 3 | 2 | 31 | 6 | 3 | 6 |

After conducting lasso regression to identify significant features, a total of 303 questions were delineated under the threshold of a beta coefficient greater than 0.2.

**Table S6.** A full list of significant questions from each cluster

** This will be presented in a separate Excel file.*

**Table S7.** SMC questionnaire for analysis (n = 80)

|  | **Category** | **Section** | **Question** | **Q-category** |
| --- | --- | --- | --- | --- |
| 1 | M.I.N.I | Major Depressive Episode | Depressed mood (Last two weeks) | depression |
| 2 | M.I.N.I | Major Depressive Episode | Diminished interest or pleasure (Last two weeks) | depression |
| 3 | M.I.N.I | Major Depressive Episode | Weight loss or gain (Last two weeks) | depression |
| 4 | M.I.N.I | Major Depressive Episode | Insomnia or hypersomnia (Last two weeks) | depression |
| 5 | M.I.N.I | Major Depressive Episode | Psychomotor agitation or retardation (Last two weeks) | depression |
| 6 | M.I.N.I | Major Depressive Episode | Fatigue or loss of energy (Last two weeks) | depression |
| 7 | M.I.N.I | Major Depressive Episode | Feelings of worthlessness or excessive or inappropriate guilt (Last two weeks) | depression |
| 8 | M.I.N.I | Major Depressive Episode | Diminished ability to think or concentrate or indecisiveness (Last two weeks) | depression |
| 9 | M.I.N.I | Major Depressive Episode | Recurrent thoughts of death or suicidal ideation (Last two weeks) | self-harm |
| 10 | M.I.N.I | Major Depressive Episode | Number of symptoms (Last two weeks) | depression |
| 11 | M.I.N.I | Major Depressive Episode | Insomnia or hypersomnia (Current) | depression |
| 12 | M.I.N.I | Major Depressive Episode | Fatigue or loss of energy (Current) | depression |
| 13 | M.I.N.I | Major Depressive Episode | Diminished ability to think or concentrate or indecisiveness (Current) | depression |
| 14 | M.I.N.I | Major Depressive Episode | Recurrent thoughts of death or suicidal ideation (Current) | self-harm |
| 15 | M.I.N.I | Major Depressive Episode | Depressed mood (Past) | depression |
| 16 | M.I.N.I | Major Depressive Episode | Recurrent thoughts of death or suicidal ideation (Past) | self-harm |
| 17 | M.I.N.I | Suicidal Tendency | Recurrent thoughts of death (Lifetime) | self-harm |
| 18 | M.I.N.I | Suicidal Tendency | Suicidal ideation (Lifetime) | self-harm |
| 19 | M.I.N.I | Suicidal Tendency | Suicidal plan (Lifetime) | self-harm |
| 20 | M.I.N.I | Suicidal Tendency | Suicide attempt (Lifetime) | self-harm |
| 21 | Clinician Rating Scales | Hamilton Rating Scale for Depression | Depressed mood | depression |
| 22 | Clinician Rating Scales | Hamilton Rating Scale for Depression | Feelings of guilt | depression |
| 23 | Clinician Rating Scales | Hamilton Rating Scale for Depression | Suicide attempt (Lifetime) | self-harm |
| 24 | Clinician Rating Scales | Hamilton Rating Scale for Depression | Insomnia early | depression |
| 25 | Clinician Rating Scales | Hamilton Rating Scale for Depression | Insomnia middle | depression |
| 26 | Clinician Rating Scales | Hamilton Rating Scale for Depression | Insomnia late | depression |
| 27 | Clinician Rating Scales | Hamilton Rating Scale for Depression | Work and activities | depression |
| 28 | Clinician Rating Scales | Hamilton Rating Scale for Depression | Retardation | depression |
| 29 | Clinician Rating Scales | Hamilton Rating Scale for Depression | Agitation | anxiety |
| 30 | Clinician Rating Scales | Hamilton Rating Scale for Depression | Anxiety | anxiety |
| 31 | Clinician Rating Scales | Hamilton Rating Scale for Depression | Anxiety-somatic | anxiety |
| 32 | Clinician Rating Scales | Hamilton Rating Scale for Depression | Somatic symptoms | depression |
| 33 | Clinician Rating Scales | Hamilton Rating Scale for Depression | Somatic symptoms gastrointestinal | depression |
| 34 | Clinician Rating Scales | Hamilton Rating Scale for Depression | Genital symptoms | depression |
| 35 | Clinician Rating Scales | Hamilton Rating Scale for Depression | Hypochondriasis | anxiety |
| 36 | Clinician Rating Scales | Hamilton Rating Scale for Depression | Weight loss | depression |
| 37 | Clinician Rating Scales | Hamilton Rating Scale for Depression | Insight | depression |
| 38 | Clinician Rating Scales | Hamilton Rating Scale for Depression | Total score | depression |
| 39 | Clinician Rating Scales | Hamilton Rating Scale for Anxiety | Anxious mood | anxiety |
| 40 | Clinician Rating Scales | Hamilton Rating Scale for Anxiety | Tension | anxiety |
| 41 | Clinician Rating Scales | Hamilton Rating Scale for Anxiety | Fears | anxiety |
| 42 | Clinician Rating Scales | Hamilton Rating Scale for Anxiety | Insomnia | anxiety |
| 43 | Clinician Rating Scales | Hamilton Rating Scale for Anxiety | Difficulties in concentration and memory | anxiety |
| 44 | Clinician Rating Scales | Hamilton Rating Scale for Anxiety | Depressed mood | depression |
| 45 | Clinician Rating Scales | Hamilton Rating Scale for Anxiety | General somatic symptoms muscular | anxiety |
| 46 | Clinician Rating Scales | Hamilton Rating Scale for Anxiety | General somatic symptoms sensory | anxiety |
| 47 | Clinician Rating Scales | Hamilton Rating Scale for Anxiety | Cardiovascular symptoms | anxiety |
| 48 | Clinician Rating Scales | Hamilton Rating Scale for Anxiety | Respiratory symptoms | anxiety |
| 49 | Clinician Rating Scales | Hamilton Rating Scale for Anxiety | Gastrointestinal symptoms | anxiety |
| 50 | Clinician Rating Scales | Hamilton Rating Scale for Anxiety | Genitourinary symptoms | anxiety |
| 51 | Clinician Rating Scales | Hamilton Rating Scale for Anxiety | Other autonomic symptoms | anxiety |
| 52 | Clinician Rating Scales | Hamilton Rating Scale for Anxiety | Behavior during interview | anxiety |
| 53 | Clinician Rating Scales | Hamilton Rating Scale for Anxiety | Total score | anxiety |
| 54 | Self-Report | Anxiety Sensitivity Index-3 (Baseline) | Physical concerns | anxiety |
| 55 | Self-Report | Anxiety Sensitivity Index-3 (Baseline) | Social concerns | anxiety |
| 56 | Self-Report | Anxiety Sensitivity Index-3 (Baseline) | Cognitive concerns | anxiety |
| 57 | Self-Report | Anxiety Sensitivity Index-3 (Baseline) | Total score | anxiety |
| 58 | Self-Report | Albany Panic and Phobia Questionnaire (Baseline) | Agoraphobia | anxiety |
| 59 | Self-Report | Albany Panic and Phobia Questionnaire (Baseline) | Social phobia | anxiety |
| 60 | Self-Report | Albany Panic and Phobia Questionnaire (Baseline) | Interoceptive | anxiety |
| 61 | Self-Report | Albany Panic and Phobia Questionnaire (Baseline) | Total score | anxiety |
| 62 | Self-Report | Anxiety Sensitivity Index-3 (Current) | Physical concerns | anxiety |
| 63 | Self-Report | Anxiety Sensitivity Index-3 (Current) | Social concerns | anxiety |
| 64 | Self-Report | Anxiety Sensitivity Index-3 (Current) | Cognitive concerns | anxiety |
| 65 | Self-Report | Anxiety Sensitivity Index-3 (Current) | Total score | anxiety |
| 66 | Self-Report | Albany Panic and Phobia Questionnaire (Current) | Agoraphobia | anxiety |
| 67 | Self-Report | Albany Panic and Phobia Questionnaire (Current) | Social phobia | anxiety |
| 68 | Self-Report | Albany Panic and Phobia Questionnaire (Current) | Interoceptive | anxiety |
| 69 | Self-Report | Albany Panic and Phobia Questionnaire (Current) | Total score | anxiety |
| 70 | Self-Report | Beck Depression Inventory-II (Baseline) | Total score | depression |
| 71 | Self-Report | Beck Hopelessness Scale (Baseline) | Total score | depression |
| 72 | Self-Report | Mood Disorder Questionnaire (Baseline) | Total score | (hypo)manic /bipolar |
| 73 | Self-Report | Hypomanic Symptom Checklist 32 (Baseline) | Total score | (hypo)manic /bipolar |
| 74 | Self-Report | Beck Anxiety Inventory (Baseline) | Total score | anxiety |
| 75 | Self-Report | Penn State Worry Questionnaire (Baseline) | Total score | anxiety |
| 76 | Self-Report | Liebowitz Social Anxiety Scale (Baseline) | Total score | anxiety |
| 77 | Self-Report | Obsessive-Compulsive Inventory-Revised (Baseline) | Total score | anxiety |
| 78 | Self-Report | Beck Depression Inventory-II (Current) | Total score | depression |
| 79 | Self-Report | Beck Hopelessness Scale (Current) | Total score | depression |
| 80 | Self-Report | Beck Anxiety Inventory (Current) | Total score | anxiety |

**Figure S1.** Characteristics of UKB cohort

|  | **All** | **Female** | **Male** |
| --- | --- | --- | --- |
| **N** | 157,348 (100.0%) | 89,089  (56.62%) | 68,259 (43.38%) |
| **Age**  (mean, std) | 38–72  (55.93, 7.74) | 40–70  (55.45, 7.66) | 38–72  (56.56, 7.8) |
| **Self-reported Dx for mental disorders** |  |  |  |
| Depression | 33,418 (21.24%) | 22,737 (25.52%) | 10,681 (15.65%) |
| Anxiety, nervousness, or generalized anxiety disorder | 22,033 (14.0%) | 14,760 (16.57%) | 7,273 (10.66%) |
| Panic attacks | 8,704 (5.53%) | 6,030 (6.77%) | 2,674 (3.92%) |
| Any other phobia (e.g., disabling fear of heights or spiders) | 2,153 (1.37%) | 1,488 (1.67%) | 665 (0.97%) |
| Social anxiety or social phobia | 1,962 (1.25%) | 1,060 (1.19%) | 902 (1.32%) |
| Obsessive compulsive disorder | 982 (0.62%) | 591 (0.66%) | 391 (0.57%) |
| Anorexia nervosa | 891 (0.57%) | 849 (0.95%) | 42 (0.06%) |
| Mania, hypomania, bipolar or manic-depression | 837 (0.53%) | 447 (0.5%) | 390 (0.57%) |
| Psychological over-eating or binge-eating | 706 (0.45%) | 600 (0.67%) | 106 (0.16%) |
| Any other type of psychosis or psychotic illness | 604 (0.38%) | 364 (0.41%) | 240 (0.35%) |
| Agoraphobia | 599 (0.38%) | 468 (0.53%) | 131 (0.19%) |
| Bulimia nervosa | 503 (0.32%) | 482 (0.54%) | 21 (0.03%) |
| Personality disorder | 385 (0.24%) | 185 (0.21%) | 200 (0.29%) |
| Autism, Asperger’s, or autistic spectrum disorder | 223 (0.14%) | 67 (0.08%) | 156 (0.23%) |
| Schizophrenia | 157 (0.1%) | 54 (0.06%) | 103 (0.15%) |
| Attention deficit or attention deficit and hyperactivity disorder | 133 (0.08%) | 66 (0.07%) | 67 (0.1%) |

**Figure S2.** Prevalence of mental disorders by self-reported diagnosis and symptom-based diagnosis

|  | **Self-reported Dx** | **Symptom-based Dx** | **overlap** |
| --- | --- | --- | --- |
| **Depression** | 33,418 (21.24%) | 37,426 (23.79%) | 20,709 (13.16%) |
| **Anxiety disorder** | 27,643 (17.57%) | 11,108 (7.06%) | 6,393 (4.06%) |
| **Bipolar disorder** | 837 (0.53%) | 2,396 (1.52%) | 391 (0.25%) |
| **Psychotic disorder** | 723 (0.46%) | 7,803 (4.96%) | 458 (0.29%) |


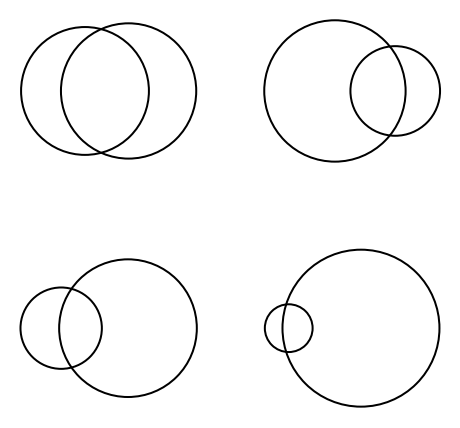


**Depression**

**Psychotic disorder**

**Bipolar disorder**

**Anxiety disorder**

7,345

458

265

2,005

391

446

16,717

20,709

12,709

4,715

6,393

21,250

SR

SB

SR

SB

SR

SB

SR

SB

Abbreviation

SR : Self-Reported Dx (left circle)

SB : Symptom-Based Dx (right circle)

Comparison of patient numbers between self-reported diagnosis and symptom-based diagnosis for each mental disorder

**Figure S3.** Distribution of disease combinations and comparison between self-reported Dx and symptom-based Dx

|  | **Self-reported Dx** | **Symptom-based Dx** |
| --- | --- | --- |
| **none** | 108,451 (68.92%) | 112,757 (71.66%) |
| **DEP** | 20,547 (13.06%) | 25,957 (16.50%) |
| **DEP-ANX** | 12,002 (7.63%) | 6,717 (4.27%) |
| **ANX** | 15,011 (9.54%) | 2,316 (1.47%) |
| **PSY** | 97 (0.06%) | 3,768 (2.39%) |
| **DEP-PSY** | 123 (0.08%) | 2,140 (1.36%) |
| **DEP-ANX-PSY** | 223 (0.14%) | 1,062 (0.67%) |
| **DEP-BIP** | 165 (0.10%) | 668 (0.42%) |
| **BIP** | 205 (0.13%) | 606 (0.39%) |
| **DEP-ANX-BIP** | 214 (0.14%) | 435 (0.28%) |
| **DEP-ANX-BIP-PSY** | 96 (0.06%) | 227 (0.14%) |
| **ANX-PSY** | 57 (0.04%) | 235 (0.15%) |
| **DEP-BIP-PSY** | 48 (0.03%) | 220 (0.14%) |
| **BIP-PSY** | 69 (0.04%) | 124 (0.08%) |
| **ANX-BIP** | 30 (0.02%) | 89 (0.06%) |
| **ANX-BIP-PSY** | 10 (0.01%) | 27 (0.02%) |

**Figure S3 (continued).** Distribution of disease combinations and comparison between self-reported Dx and symptom-based Dx


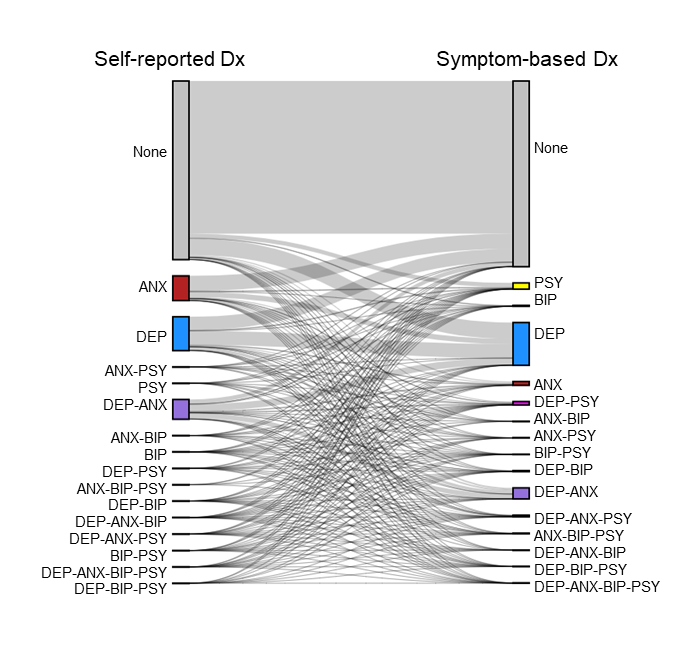


Overall, 52,366 (33.28%) samples were discordant between Self-reported Dx and Symptom-based Dx.

**Figure S4.** Frequency of the most frequently prescribed drugs in each drug class


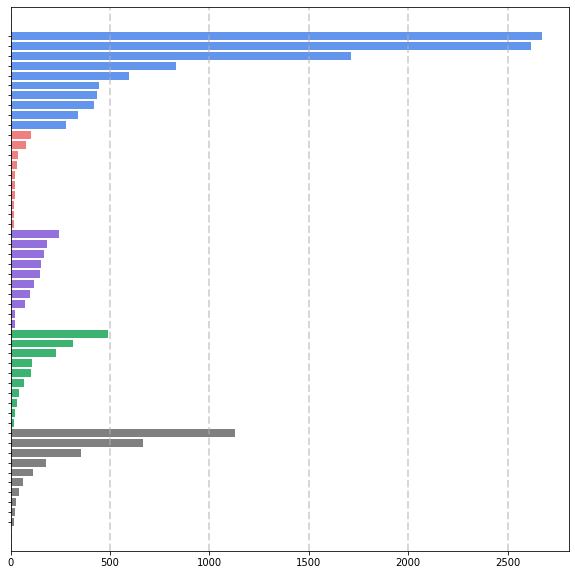


citalopram

amitriptyline

fluoxetine

sertraline

venlafaxine

paroxetine

mirtazapine

dosulepin

st john's wort/hypericum [ctsu]

escitalopram

olanzapine

quetiapine

risperidone

chlorpromazine

trifluoperazine

seroquel 25mg tablet

sulpiride

haloperidol

aripiprazole

amisulpride

tegretol 100mg

lamotrigine

carbamazepine

epilim 100mg crushable

sodium valproate

lithium product

topiramate

priadel 200mg m/r tablet

depakote 250mg e/c tablet

lamictal 25mg tablet

zopiclone

diazepam

temazepam

clonazepam

zolpidem

nitrazepam

lorazepam

zimovane ls 3.75mg tablet

valium 2mg tablet

clobazam

propranolol

gabapentin

pregabalin

clonidine

ropinirole

nicotine product

inderal 10mg tablet

clonidine hydrochloride 25mg

procyclidine

trihexyphenidyl

frequency

0

500

1000

1500

2000

2500

Anti Depressant (AD)

Anti Psychotics (AP)

Mood Stabilizer (MS)

Sedative-Hypnotic drug (SH)

etc

**Figure S5.** Drug prescription information of 14,358 individuals in UKB cohort.


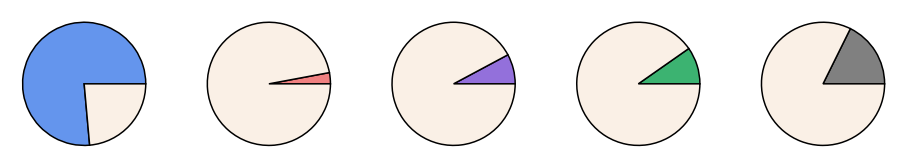


AD

AP

MS

SH

etc

76.42

2.93

7.82

9.72

17.68


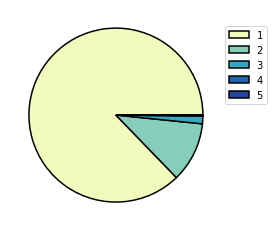


87.24

11.15

1.42

**A**

**B**

1

2

3

4

5

Number of drug classes prescribed

(per person)

0.17

0.01

(A) Distribution of the number of drug classes prescribed per person. (B) Proportion of people prescribed with each drug class.

**Figure S6.** Overview of the network-based recommendation system

Number of neighbors: 20
Number of neighbors with trt: 11 (55 %)

target sample

Neighbor /w trt info

Neighbor /wo trt info

(AP) x 5

(MS) x 1

(SH) x 1

(AD) x 4

(i) Establish the initial neighborhood by selecting individuals most similar to a target patient within the pre-constructed network, generated from community detection covering 157,348 individuals. Because only approximately 9% (14,358) individuals had accessible information regarding drug prescriptions, some of these neighbors may have information on drug prescriptions, while others may not.

(ii) Expand the neighborhood gradually, to ensure sufficient drug prescription data within the neighborhood, requiring at least N individuals with accessible drug prescription information. In this study, we set this number to 20 individuals, and this criterion is adjustable and depends on the severity of mental illness and availability of drug prescription history.

(iii) Finalize drug recommendations based on the information obtained from the final neighborhood, compiling a list of recommended drugs ordered by prescription frequency. The final recommendations comprised drug classes with prescription rankings exceeding a predetermined threshold.

**Figure S7.** Results of network-based drug recommendation including cluster information

100

80

60

40

20

0

100

80

60

40

20

0

60

50

40

30

20

10

0

100

80

60

40

20

0

70

60

50

40

30

20

10

0

(%)

(%)

(%)

(%)

(%)

Recommended

Top 1

Top 3

Top 5

Top 10

Prescribed

Actual

**AD**

**AP**

**MS**

**SH**

**etc**


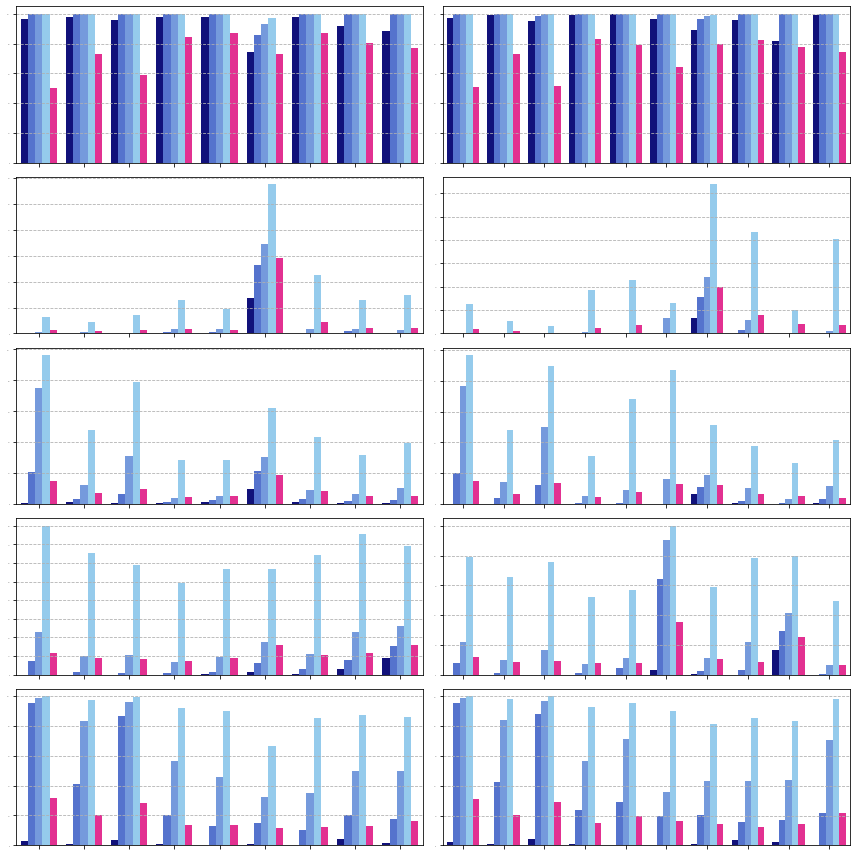


KM0

KM4

KM8

LV0

LV4

LV6

LV2

LV8

LV1

LV5

LV7

LV3

LV9

KM1

KM5

KM2

KM6

KM3

KM7

The four bars (bluish) on the left represent the proportion of people who received recommendations for each drug class in each cluster applying several thresholds, i.e., 1, 3, 5, and 10. The rightmost pink bar represents the percentage of patients who were actually prescribed each drug class. (Left) KM clustering. (Right) LV clustering.


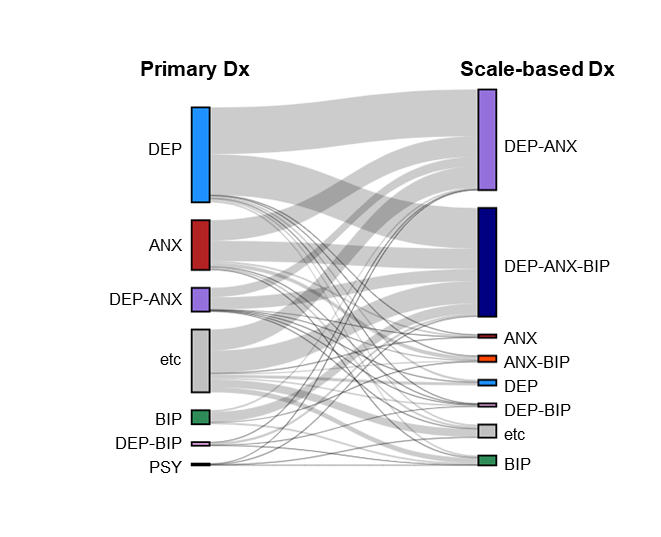
**Figure S8.** Distribution of disease combinations and comparisons between self-reported Dx and symptom-based Dx in SMC cohort

|  | **Primary Dx** | **Scale-based Dx** |
| --- | --- | --- |
| **DEP** | 319 (37.89%) | 19 (2.26%) |
| **etc** | 211 (25.06%) | 44 (5.32%) |
| **ANX** | 167 (19.83%) | 11 (1.31%) |
| **DEP-ANX** | 80 (9.50%) | 338 (40.14%) |
| **BIP** | 47 (5.58%) | 33 (3.92%) |
| **DEP-BIP** | 12 (1.43%) | 11 (1.31%) |
| **PSY** | 6 (0.71%) | 0 (0%) |
| **DEP-ANX-BIP** | 0 (0%) | 365 (43.35%) |
| **ANX-BIP** | 0 (0%) | 21 (2.49%) |
| **total** | 842 (100%) | |

(Left) The table reveals the prevalence of mental disorders of 842 patients in SMC cohort based on Primary Dx and Scale-based Dx, respectively. (Right) Primary Dx and Scale-based Dx were compared using Sankey plot.

**Figure S9.** Information on drug prescription in SMC cohort.

**A**


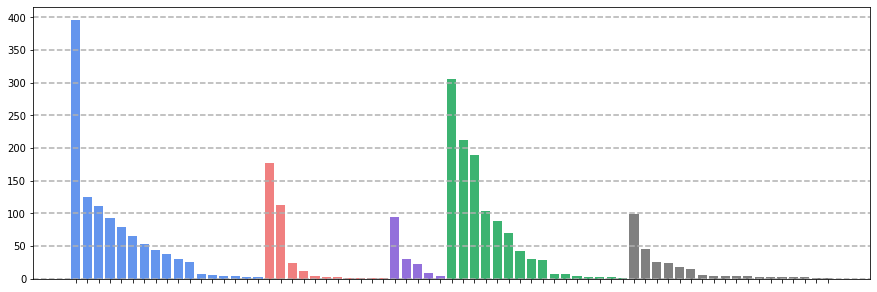


escitalopram

mirtazapine

paroxetine

duloxetine

desvenlafaxine

venlafaxine

vortioxetine

fluoxetine

bupropion

sertraline

tianeptine

milnacipran

agomelatine

amitriptyline

nortriptyline

fluvoxamine

imipramine

quetiapine

aripiprazole

olanzapine

risperidone

paliperidone

haloperidol

sulpiride

amisulpride

blonanserin

clozapine

ziprasidone

divalproex

lithium

lamotrigine

valproate

topiramate

alprazolam

lorazepam

clonazepam

zolpidem

trazodone

etizolam

melatonin

diazepam

doxepine

clobazam

loflazepate

clomipramine

triazolam

chlordiazepoxide

tofisopam

eszopiclone

propranolol

choline

buspirone

acamprosate

methylphenidate

naltrexone

benztropine

donepezil

pregabalin

lorcaserin

atomoxetine

memantine

gabapentin

ropinirole

phentermine

rivastigmine

procyclidine

contrave

frequency

350

300

250

200

150

100

50

0

400

Anti Depressant (AD)

Anti Psychotics (AP)

Mood Stabilizer (MS)

Sedative-Hypnotic drug (SH)

etc

**B**

**C**


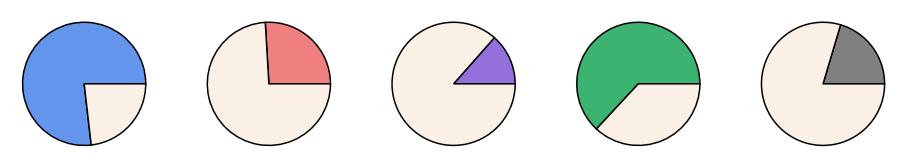


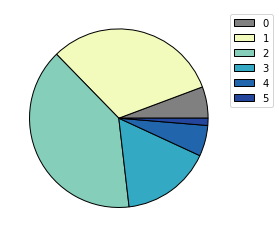


31.59

39.55

16.27

5.7

1.31

5.58

0

1

2

3

4

5

Number of drug classes prescribed

(per person)

AD

AP

MS

SH

etc

76.83

25.94

13.48

63.1

20.4

(A) Prescription rate of the most frequently prescribed drugs in each drug class. (B) Pie chart presents the distribution of the number of drug classes prescribed per person. (C) The pie chart shows the proportion of people prescribed with drugs from each drug class.

**Figure S10.** Statistics for cluster-based drug recommendation in SMC cohort.


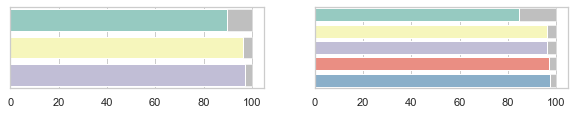


0

20

40

60

80

100

(%)

0

20

40

60

80

100

(%)

KM0

KM1

KM2

LV0

LV1

LV3

LV2

LV4

Percentile of samples with a history of psychiatric drugs for each cluster identified by KM (left) and LV (right) methods.

**Figure S11.** Results of network-based drug recommendation including cluster information


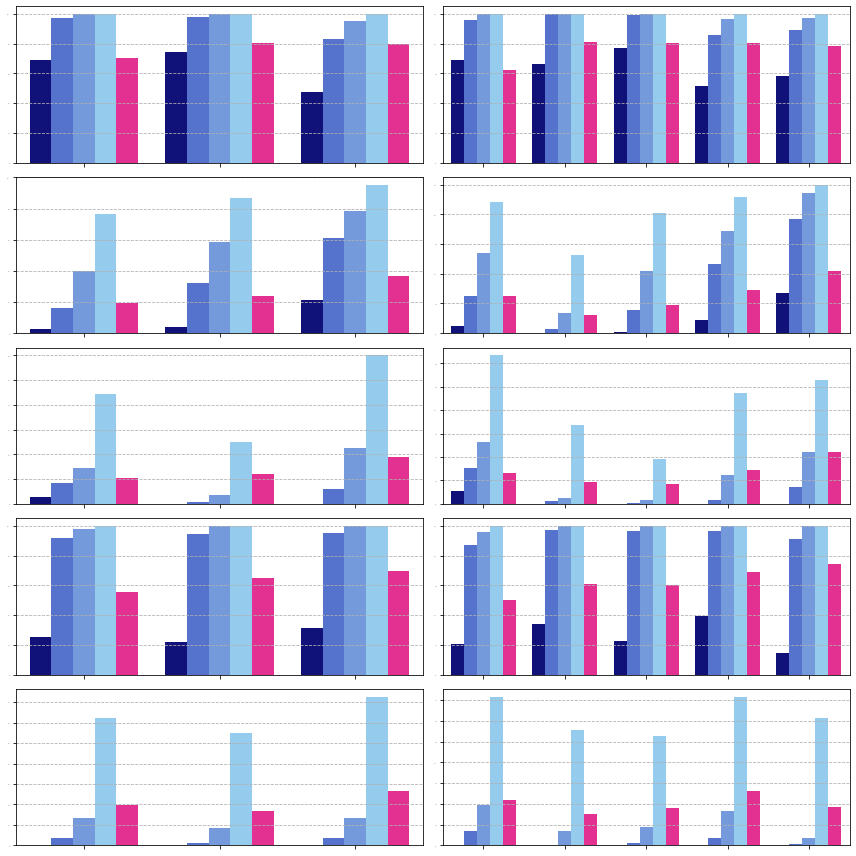


KM0

KM1

KM2

LV0

LV2

LV3

LV1

LV4

100

80

60

40

20

0

100

80

60

40

20

0

60

50

40

30

20

10

0

100

80

60

40

20

0

70

60

50

40

30

20

10

0

(%)

(%)

(%)

(%)

(%)

Recommended

Top 1

Top 3

Top 5

Top 10

Prescribed

Actual

**AD**

**AP**

**MS**

**SH**

**etc**

The four bars (bluish) on the left represent the proportion of people who received recommendations for each drug class in each cluster applying several thresholds, i.e., 1, 3, 5, and 10. The rightmost pink bar represents the percentage of patients who were actually prescribed with each drug class. (Left) KM clustering. (Right) LV method.
